# Ubiquitin-like protein 5 is a novel player in the UPR–PERK arm and ER stress–induced cell death

**DOI:** 10.1016/j.jbc.2023.104915

**Published:** 2023-06-12

**Authors:** Wei Wang, Adam M. Hawkridge, Yibao Ma, Bei Zhang, John B. Mangrum, Zaneera H. Hassan, Tianhai He, Sofiya Blat, Chunqing Guo, Huiping Zhou, Jinze Liu, Xiang-Yang Wang, Xianjun Fang

**Affiliations:** 1 Department of Biochemistry & Molecular Biology, School of Medicine, Virginia Commonwealth University, Richmond, Virginia, USA; 2 Department of Pharmaceutics, School of Pharmacy, Virginia Commonwealth University, Richmond, Virginia, USA; 3 Department of Biostatistics, School of Medicine, Virginia Commonwealth University, Richmond, Virginia, USA; 4 Department of Human & Molecular Genetics, School of Medicine, Virginia Commonwealth University, Richmond, Virginia, USA; 5 Department of Microbiology & Immunology, School of Medicine, Virginia Commonwealth University, Richmond, Virginia, USA; 6 Hunter Holmes McGuire VA Medical Center, Richmond, Virginia, USA

**Keywords:** UBL5, ER stress, UPR, PERK, proteasome degradation, ubiquitin-independent proteasome system, apoptosis, cell survival

## Abstract

Biological functions of the highly conserved ubiquitin-like protein 5 (UBL5) are not well understood. In *Caenorhabditis elegans*, UBL5 is induced under mitochondrial stress to mount the mitochondrial unfolded protein response (UPR). However, the role of UBL5 in the more prevalent endoplasmic reticulum (ER) stress-UPR in the mammalian system is unknown. In the present work, we demonstrated that UBL5 was an ER stress–responsive protein, undergoing rapid depletion in mammalian cells and livers of mice. The ER stress–induced UBL5 depletion was mediated by proteasome-dependent yet ubiquitin-independent proteolysis. Activation of the protein kinase R–like ER kinase arm of the UPR was essential and sufficient for inducing UBL5 degradation. RNA-Seq analysis of UBL5-regulated transcriptome revealed that multiple death pathways were activated in UBL5-silenced cells. In agreement with this, UBL5 knockdown induced severe apoptosis in culture and suppressed tumorigenicity of cancer cells *in vivo*. Furthermore, overexpression of UBL5 protected specifically against ER stress–induced apoptosis. These results identify UBL5 as a physiologically relevant survival regulator that is proteolytically depleted by the UPR-protein kinase R–like ER kinase pathway, linking ER stress to cell death.

Ubiquitin binds to cellular proteins *via* an isopeptide bond between the two glycine (Gly) residues (di-Gly motif) at its C terminus and an acceptor lysine residue of target proteins catalyzed by the sequential activities of ubiquitin-activating enzymes (E1), ubiquitin-conjugating enzymes (E2), and ubiquitin protein ligases (E3) (1, 2). Attachment of ubiquitin subsequently alters the stabilities, localizations, or activities of target proteins. In addition to ubiquitin, there exist a large number of ubiquitin-like proteins (UBLs) that are related in the sequence, structure, and function to ubiquitin (3, 4). The members of UBLs share a canonical ubiquitin fold composed of a curved β-sheet wrapping around a central α-helix. Many UBLs such as small ubiquitin-related modifier and neural precursor cell expressed, developmentally downregulated 8 also contain the di-Gly motif responsible for target conjugation through the E1–E3 enzymatic cascade (4), adding another dimension of covalent modifications of protein functions.

Among UBLs, the highly conserved ubiquitin-like protein 5 (UBL5)/homologous to ubiquitin 1 is unique in that it lacks the C-terminal di-Gly motif necessary to form covalent conjugates with other proteins (5, 6, 7). Instead, UBL5 possesses a C-terminal dityrosine motif of unknown function. As such, UBL5 is thought to be a reversible regulator of protein functions rather than a protein degrader. Biological functions of UBL5, particularly in mammalian cells, remain poorly understood. UBL5 has been shown to regulate pre-mRNA splicing *via* noncovalent binding to specific spliceosomal proteins in yeast and human cells (8, 9, 10, 11, 12, 13). UBL5 was also reported to be present in the Fanconi anemia complementation group I to maintain the functional integrity of the Fanconi anemia DNA repair pathway in mammalian cells (14). In *Caenorhabditis elegans*, UBL5 was induced by mitochondrial stress to mount the mitochondrial unfolded protein response (UPR^mt^) (15). The induced UBL5 binds to the transcription factor Dve-1 to activate expression of the mitochondrial chaperone hsp-60 and other UPR^mt^ components (15, 16, 17). Recent studies also implicate UBL5 in activation of the UPR^mt^ in mammalian cells (https://minerva-access.unimelb.edu.au/items/2f2fccf6-655e-5e34-bedb-8c7dd2a04b9b, Accessed June 29, 2022) (18). However, the role of UBL5 in the physiologically more prevailing endoplasmic reticulum (ER) stress and associated UPR is unknown.

In the present study, we examined the role of UBL5 in ER stress-UPR in the mammalian system. We demonstrated that UBL5 protein was quickly lost in response to diverse ER stressors in mammalian cell lines and in livers of mice in sharp contrast to the observation in *C. elegans* where UBL5 was induced upon mitochondrial stress (15). The degradation of UBL5 in response to ER stress was mediated by the ubiquitin-independent proteasome system (UIPS) (19). Pharmacological and molecular analyses indicate that the protein kinase R–like ER kinase (PERK) of the UPR was essential and sufficient for inducing UBL5 degradation in response to ER stress. The depletion of UBL5 led to activation of multiple death cascades as revealed by RNA-Seq and pathway enrichment analysis. Consistent with this, UBL5 deficiency induced catastrophic apoptosis in culture and inhibited tumorigenicity of cancer cells *in vivo*. Furthermore, overexpression of UBL5 was capable of conferring significant resistance to apoptosis induced by ER stress but not by other death stimuli unaffecting UBL5 protein stability. These results demonstrate a physiologically relevant survival function of UBL5 and a novel regulatory mechanism to control UBL5 protein stability by activation of PERK during ER stress.

## Results

### UBL5 protein is depleted as a ubiquitous response to ER stress

**Figure 1:**
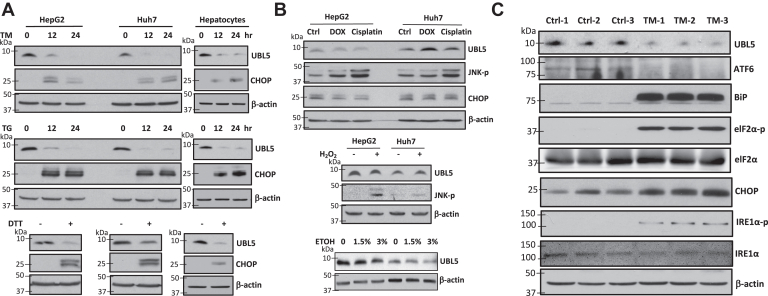
**Loss of UBL5 is a general response to ER stress.***A*, HepG2, Huh7, and primary mouse hepatocytes were treated with TM (2, 0.2, 0.5 μg/ml, respectively), TG (1, 0.1, and 0.25 μM, respectively) for indicated hours or with DTT (1 mM, 3 h in all cell types). In this and the following panels, expression of UBL5 protein was analyzed with tricine protein gel. CHOP was included as a UPR activation marker and β-actin as loading control. *B*, HepG2 and Huh7 cells were treated with DOX (0.4 μM, 16 h), cisplatin (7.5 μM, 16 h), H_2_O_2_ (0.4 mM, 24 h), or ethanol (ETOH) (1.5% or 3%, 24 h) before immunoblotting analysis of indicated proteins. *C*, adult C57/BL6 male mice were i.p. injected with vehicle or with TM (2 mg/kg) (n = 3) to induce pharmacological ER stress. After 26 h, the control and TM-injected mice were sacrificed and liver tissues were collected for protein extraction. The effects of TM treatment on expression of UBL5 protein and multiple UPR activation markers (ATF6, BiP, elF2α-p, elF2α, CHOP, IRE1α-p, and IRE1α) were assessed by immunoblotting. ATF6, activating transcription factor 6; BiP, binding immunoglobulin protein; CHOP, C/EBP homologous protein; DOX, doxorubicin; ER, endoplasmic reticulum; H_2_O_2_, hydrogen peroxide; IRE1α, inositol-requiring enzyme 1α; TG, thapsigargin; TM, tunicamycin; UBL5, ubiquitin-like protein 5.

Little is known about how expression of UBL5 is regulated in physiological and pathophysiological conditions. To address this, we examined UBL5 protein expression in the context of ER stress in mammalian cell lines and primary mouse hepatocytes. In regular culture, the approximately 8.5 kDa UBL5 protein was constitutively present in all cell lines of different tissue origins as analyzed with immunoblotting (data not shown). However, the protein disappeared in response to ER stress–inducing agents such as the most commonly used pharmacological ER stressors tunicamycin (TM), thapsigargin (TG), and DTT (Fig. 1*A*). When TM or TG was withdrawn from culture, UBL5 protein did not recover immediately until clearance of ER stress as reflected by termination of C/EBP homologous protein (CHOP) induction or PERK activation (Fig. S1), confirming the close association of UBL5 depletion with ER stress condition.

Different from TM, TG, and DTT, certain cellular insults do not cause ER stress *per se* but could disturb ER homeostasis ultimately leading to ER stress. To test whether these indirect ER stimuli regulate UBL5 protein abundance, we examined the responses of UBL5 to inhibitors of electron transfer chains or hypoxia. Electron transfer chain inhibitors have been reported to interfere with ATP-dependent protein folding secondary to the onset of bioenergetic stress (20, 21). Indeed, treatment for 24 h with the complex I inhibitor rotenone or piericidin A blocked ATP production as reflected by phosphorylation and activation of AMP-activated kinase, which was accompanied with marked depletion of UBL5 protein (Fig. S1). Similarly, the antidiabetic agent metformin at 2 mM also resulted in partial loss of UBL5 protein, consistent with its reported role as a complex I inhibitor at high doses (22). Hypoxia also induced loss of UBL5 (Fig. S2), consistent with the roles of oxygen in formation of disulfide linkages or in ATP production required for protein folding (23).

In contrast to these direct and indirect ER stress inducers, the chemotherapeutic agents doxorubicin (DOX) and cisplatin did not affect UBL5 protein abundance although they all induced general cellular stress as evidenced by phosphorylation and activation of c-Jun N-terminal kinase (Fig. 1*B*). However, they did not induce ER stress as reflected by the lack of CHOP induction (Fig. 1*B*). Exposure of HepG2 and Huh7 cells to ethanol or hydrogen peroxide (H_2_O_2_) for 24 h did not deplete UBL5 either, confirming that UBL5 was depleted in response to ER stress but not other cellular stress conditions.

To confirm that UBL5 protein was also depleted in response to ER stress *in vivo*, we took advantage of the TM-induced ER stress model (24). Mice were i.p. injected with TM (2 mg/kg) or vehicle. Fractions of livers were collected and processed for immunoblotting. As shown in Figure 1*C*, TM treatment induced full-fledged UPR as indicated by activation of multiple markers of the three UPR arms: activating transcription factor 6 (ATF6), binding immunoglobulin protein, phosphorylated eukaryotic translation initiation factor 2α (eIF2α), CHOP, and phosphorylated inositol-requiring enzyme 1α (IRE1α) (25). Concurrent with the UPR activation by TM, UBL5 was dramatically decreased in livers of TM-treated mice compared with those of control animals.

### Loss of UBL5 protein is mediated by the UIPS

**Figure 2:**
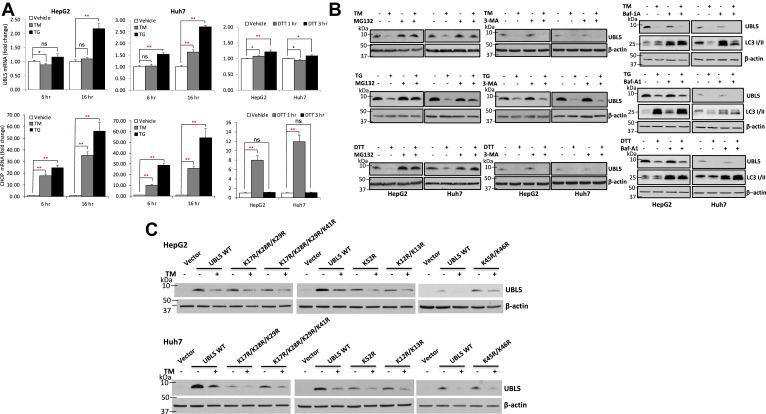
**ER stress–induced UBL5 degradation is mediated by the UIPS independent of transcriptional regulation.***A*, HepG2 and Huh7 cells were treated with TM or TG for 6 or 16 h or DTT for 1 or 3 h at doses as for Figure 1*A*. The effects on UBL5 mRNA were analyzed by RT–qPCR. The results were normalized on that of β-actin and presented as fold change relative to vehicle-treated control cells (defined as 1). The statistical significances were indicated with *black* and *red asterisks* to indicate upregulation and downregulation, respectively. *B*, HepG2 and Huh7 cells were treated with TM or TG for 16 h at doses as for Figure 1*A* in the presence or the absence of MG132 (1 μM), 3-MA (5 mM), or Baf-A1 (25 nM). The effects of these treatments on UBL5 protein were examined by immunoblotting. *C*, tag-free wildtype UBL5 (UBL5 wt) or various lysine/arginine (A/R) mutants were transiently transfected into HepG2 or Huh7 cells. Transfected cells were treated with TM for 20 h at doses as for Figure 1*A*. The TM-induced degradation of wt and mutant UBL5 was examined by immunoblotting. 3-MA, 3-methyladenine; Baf-A1, bafilomycin A1; ER, endoplasmic reticulum; qPCR, quantitative PCR; TG, thapsigargin; TM, tunicamycin; UBL5, ubiquitin-like protein 5; UIPS, ubiquitin-independent proteasome system.

To understand the mechanism for the loss of UBL5 protein during ER stress, we examined UBL5 mRNA expression by reverse transcription (RT) and real-time PCR. As shown in Figure 2*A*, HepH2 and Huh7 cells were treated with TM, TG, or DTT for different intervals. These treatments were sufficient to induce ER stress and UPR as evidenced by dramatic induction of expression of CHOP transcripts (Fig. 2*A*, *lower*). However, only slight decreases in UBL5 mRNA were detected in HepG2 cells treated with TM for 6 h (∼11%) and in Huh7 cells treated with DTT for 1 h (<5%), which was disproportionate to the extent of UBL5 protein loss induced by these agents. In addition, these minor decreases were not consistent between the two cell lines or between different time points. In fact, we observed significant increases in UBL5 mRNA levels in later time points of TM, TG, or DTT treatment (Fig. 2*A*), likely as a compensatory response to the loss of UBL5 protein under ER stress. Collectively, these results indicate that ER stress triggers loss of UBL5 protein through a mechanism independent of transcriptional repression.

We next examined whether the decrease in UBL5 protein under ER stress resulted from proteasome-mediated process. HepG2 and Huh7 cells were treated with TM, TG, or DTT in the absence or the presence of the proteasome inhibitor MG-132. As shown in Figure 2*B* (*left*), MG-132 at 1 μM fully or significantly rescued TM-, TG-, or DTT-induced loss of UBL5 protein. In contrast, inhibitors of the autophagic/lysosomal pathway, 3-methyladenine (26) and bafilomycin A1 (27) did not protect UBL5 protein against ER stress (Fig. 2*B*, *middle & right*), indicating the involvement of the proteasome-dependent proteolysis but not the autophagic/lysosomal pathway in degrading UBL5 in response to ER stress.

UBL5 is a lysine-rich protein with a total of nine lysine residues (7). The protein has not been shown to be regulated by the ubiquitin proteasome system. Consistently, we did not see apparent UBL5–ubiquitin ladders at early hours or over the entire course of TM treatment (Fig. S3). However, this could be due to highly dynamic degradation upon ubiquitination or the limitation of the UBL5 antibody to detect UBL5 ubiquitinated *via* different types of ubiquitin chain linkages (28, 29). Interestingly, a recent proteomics study aiming for genome-wide profiling of ubiquitin-binding proteins demonstrated that four lysine residues within UBL5 (K17, K28, K29, and K41) were ubiquitinated at basal levels in HepG2 cells (30). We were also able to detect three (K17, K28, and K29) of these four lysine residues bound to ubiquitin in UBL5-His-transfected human embryonic kidney 293 cells by mass spectrometry analysis (data not shown). However, when we mutated these three (K17R/K28R/K29R) or all four (K17R/K28R/K29R/K41R) lysine residues, TM-induced degradation of the mutants showed a pattern similar to that of wt UBL5 in HepG2 and Huh7 cells (Fig. 2*C*).

In the subsequent experiment, we mutated other five lysine residues individually for well-separated ones or collectively for those clustered together. As shown in Figure 2*C*, these additional mutants remained sensitive to TM-induced degradation. Together, none of these lysine residues seems to be required for proteasome-dependent degradation of UBL5. Therefore, ER stress–induced degradation of UBL5 is most likely mediated *via* UIPS. Of note, TM induced only partial degradation of overexpressed UBL5, compared with the endogenous UBL5, suggesting that overexpressed UBL5 exceeded the UIPS-degrading capacity in TM-treated conditions.

### UBL5 degradation lies downstream of the PERK arm of the UPR

**Figure 3:**
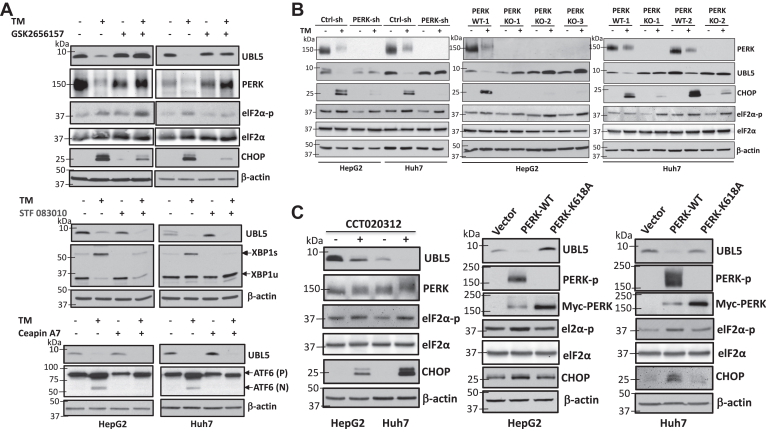
**UBL5 degradation lies downstream of the PERK arm of UPR.***A*, HepG2 and Huh7 cells were treated for 18 h with TM at doses as for Figure 1*A* in the presence or the absence of the PERK inhibitor GSK2656157 (0.12 μM and 0.25 μM, respectively), the IRE1/XBP1 pathway inhibitor STF083010 (80 μM and 50 μM, respectively), or the ATF6 blocker ceapin-A7 (12 μM for both cell lines). The effects of these treatments on UBL5 and UPR markers were examined by immunoblotting. CHOP, XBP1s (spliced form), and ATF6 cleavage were included to show the efficacies of the respective inhibitors. *B*, PERK was knocked down by shRNA (*left*) or knocked out by CRISPR–Cas9 (PERK-KO) (*right*). The cells were treated with TM for 16 h at doses as for Figure 1*A* before immunoblotting for the indicated proteins. *C*, HepG2 and Huh7 cells were treated with the PERK activator CCT020312 (8 μM, 16 h) or vehicle before immunoblotting for the indicated proteins (*left*). *Right*, Myc-tagged wt PERK (PERK-WT) or its kinase dead mutant (PERK-K618A) was transfected into HepG2 and Huh7 cells. Cell lysates were prepared 48 h post-transfection and immunoblotted for the indicated proteins. ATF6, activating transcription factor 6; CHOP, C/EBP homologous protein; PERK, protein kinase R–like ER kinase; TM, tunicamycin; UBL5, ubiquitin-like protein 5; UPR, unfolded protein response; XBP1, X-box-binding protein 1.

The aforementioned results suggested that UBL5 is a previously unrecognized ER stress–responsive protein. We next examined if UBL5 proteolysis is mediated by the UPR or by a mechanism independent of the three known UPR arms. To this end, we examined the effects of well-characterized inhibitors of three UPR sensors PERK, IRE1/X-box-binding protein 1 (XBP1), and ATF6 on UBL5 degradation. As shown in Figure 3*A*, TM-induced UBL5 degradation was prevented by the PERK inhibitor GSK265615 (31) but not by the IRE1/XBP1 inhibitor STF 083010 (32) or the ATF6 blocker ceapin-A7 (33), suggesting that UBL5 turnover is controlled by PERK activity under ER stress.

To gain molecular evidence for the involvement of PERK in the UBL5 degradation, we used virally transduced shRNA to knockdown (KD) PERK expression as well as the CRISPR–Cas9 technique to knockout PERK in HepG2 and Huh7 cells. As presented in Figure 3*B*, both approaches protected UBL5 from TM-induced degradation. Of note, UBL5 was completely protected by either PERK inhibitor GSK265615 (Fig. 3*A*) or by PERK KD or KO intervention (Fig. 3*B*). In HepG2 cells, the PERK KO was actually overprotective, resulting in higher UBL5 protein abundance in TM-treated cells than in untreated control cells (Fig. 3*B*, *right*). The close association of PERK activity with UBL5 protein degradation suggests that UBL5 stability is tightly controlled by PERK activity. In other words, UBL5 protein could be fully protected from ER stress in the absence of PERK. In contrast to UBL5 depletion, eIF-2α phosphorylation, another event downstream of PERK, was not much affected by intervention of PERK (Fig. 3*B*), in agreement with the existence of redundant or adaptively activated pathways to phosphorylate eIF-2α as reported previously (34, 35).

We next asked whether activation of PERK alone was sufficient to trigger UBL5 degradation. Indeed, the PERK-specific activator CCT020312 (36) induced PERK phosphorylation (as reflected by its mobility shift) and UBL5 degradation (Fig. 3*C*). We also overexpressed Myc-tagged wt PERK (PERK-WT) or its kinase-dead mutant (PERK-K618A) (37) to determine the effect of PERK activation on the status of UBL5 protein. As expected, overexpression of PERK-WT but not the K618A mutant was capable of inducing UBL5 degradation (Fig. 3*C*). These results together establish that activation of PERK is essential and sufficient for triggering UBL5 degradation under ER stress.

### UBL5 is located in both cytoplasm and nucleus

**Figure 4:**
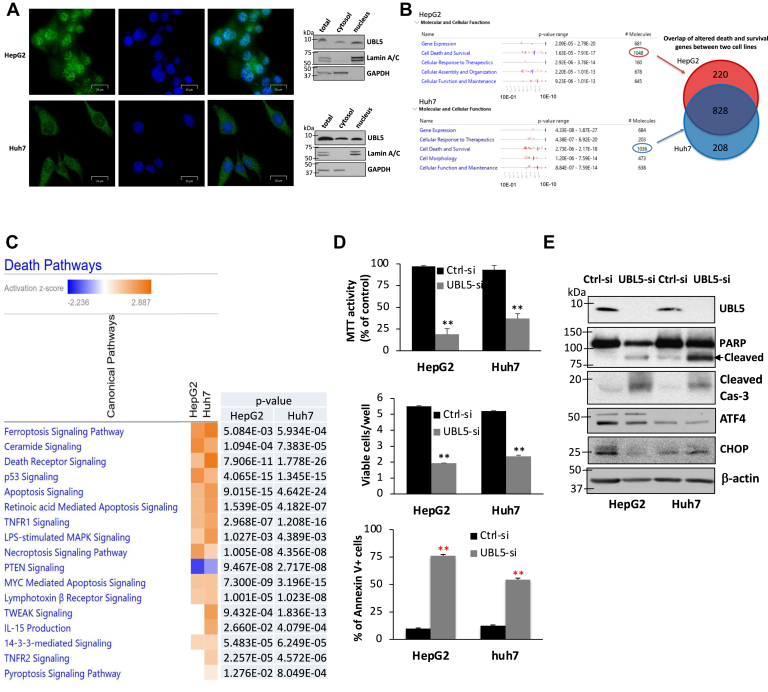
**UBL5 KD activates multiple death pathways and induces severe apoptosis.***A*, HepG2 and Huh7 cells were costained with an anti-UBL5 antibody and the blue-fluorescent DNA stain DAPI (*left*). *Right*, cytoplasmic and unclear proteins were fractionated. Total cellular lysates (total), cytosolic or nuclear fraction isolated from the same number of cells, were analyzed by immunoblotting with GAPDH and Lamin A/C as cytosolic and nuclear markers, respectively. *B*, HepG2 and Huh7 cells were transfected with Ctrl-siRNA or UBL5 siRNA. After 48 h, RNA was isolated for RNA-Seq analysis. RNA-Seq data from these cells were analyzed with IPA. Statistically significantly upregulated or downregulated protein-coding transcripts (padj < 0.05) were included for analysis and categorized into functionally related groups. Listed were functional groups that were significantly enriched in order of their *p* value ranges (low to high). The total numbers of affected transcripts in each group are listed in the *right* column. *C*, death and survival-related transcripts significantly altered in UBL5 siRNA KD cells were analyzed with the DAVID functional annotation and IPA enrichment programs. The specific death pathways activated or suppressed by siRNA KD in each cell line were shown with heatmaps in order of their *Z* scores (high to low). The statistical significances of enriched pathways are provided in *right*. *D*, HepG2 and Huh7 cells were transfected with UBL5 siRNA or Ctrl-siRNA. The transfected cells were analyzed 3 days post-transfection for viability by MTT staining (*upper*), quantification of viable cells with trypan blue exclusion (*middle*), or percentages of apoptosis with Annexin V staining (*lower*). *E*, the apoptotic markers (PARP and Cas-3 cleavage) and the effects of UBL5 siRNA on expression of ATF4 and CHOP were examined by immunoblotting. ATF4, activating transcription factor 4; CHOP, C/EBP homologous protein; DAPI, 4′,6-diamidino-2-phenylindole; IPA, ingenuity pathway analysis; KD, knockdown; MTT, 3-(4,5-dimethylthiazol-2-yl)-2,5-diphenyltetrazolium bromide; PARP, poly(ADP-ribose) polymerase; UBL5, ubiquitin-like protein 5.

There are no clear structural bases to predict subcellular localization of UBL5 protein. Previous reports of intracellular localization of UBL5 were either performed on exogenously expressed epitope-tagged UBL5 or vary in their conclusions on the locations of the endogenous UBL5 protein (16, 17, 38, 39). We examined several commercially available UBL5 antibodies and optimized immunofluorescence staining conditions for the endogenous UBL5 as detailed in the Experimental procedures section. We observed clearly stained cytoplasm and nuclei in HepG2 and Huh7 cells (Fig. 4*A*, *left*). Further subcellular fractionation analysis confirmed the presence of UBL5 protein in both cytosolic and nuclear fractions (Fig. 4*A*, *right*). These subcellular localizations suggest that UBL5 protein shuttles to regulate its stability in the cytoplasm and its function in the nucleus.

### UBL5 silencing activates multiple death pathways and induces apoptosis

We next focused on the biological significance of UBL5 depletion and regulation. Previous studies suggest that UBL5 may modulate gene expression *via* binding to a transcription factor (16, 17) or to a component of the pre-mRNA spliceosome (8, 10, 11, 12, 13). To determine the effects of UBL5 on genome-wide gene expression, we used UBL5 siRNA KD cells to conduct RNA-Seq analysis.

When all significantly upregulated and downregulated protein-coding transcripts (padj <0.05) were included for the Qiagen ingenuity pathway analysis, several large groups of functionally related genes emerged as significantly enriched by UBL5 KD in both HepG2 and Huh7 cells (Fig. 4*B*). Among them, the death and survival-related genes made the largest functional group consisting of more than 1000 related transcripts in each cell line. There was >80% overlap of these transcripts between the two cell lines (Fig. 4*B*). Other functional groups affected by UBL5 KD included those regulating gene expression, cellular responses to therapeutics, and other cellular functions and maintenance. Further functional annotation and pathway enrichment analysis revealed that most of the well-defined prodeath pathways were significantly activated by UBL5 KD in both cell lines (Fig. 4*C*). Consistent with upregulation of most prodeath pathways, UBL5 KD cells underwent catastrophic apoptosis, peaking at 3 days post-transfection as determined by expression of apoptotic markers, staining with 3-(4,5-dimethylthiazol-2-yl)-2,5-diphenyltetrazolium bromide (MTT) (40), quantification of viable cells with trypan blue exclusion or Annexin V staining (Fig. 4*D*). As a result, UBL5 siRNA KD cells were negatively selected and rapidly eliminated from continuous culture.

**Figure 5:**
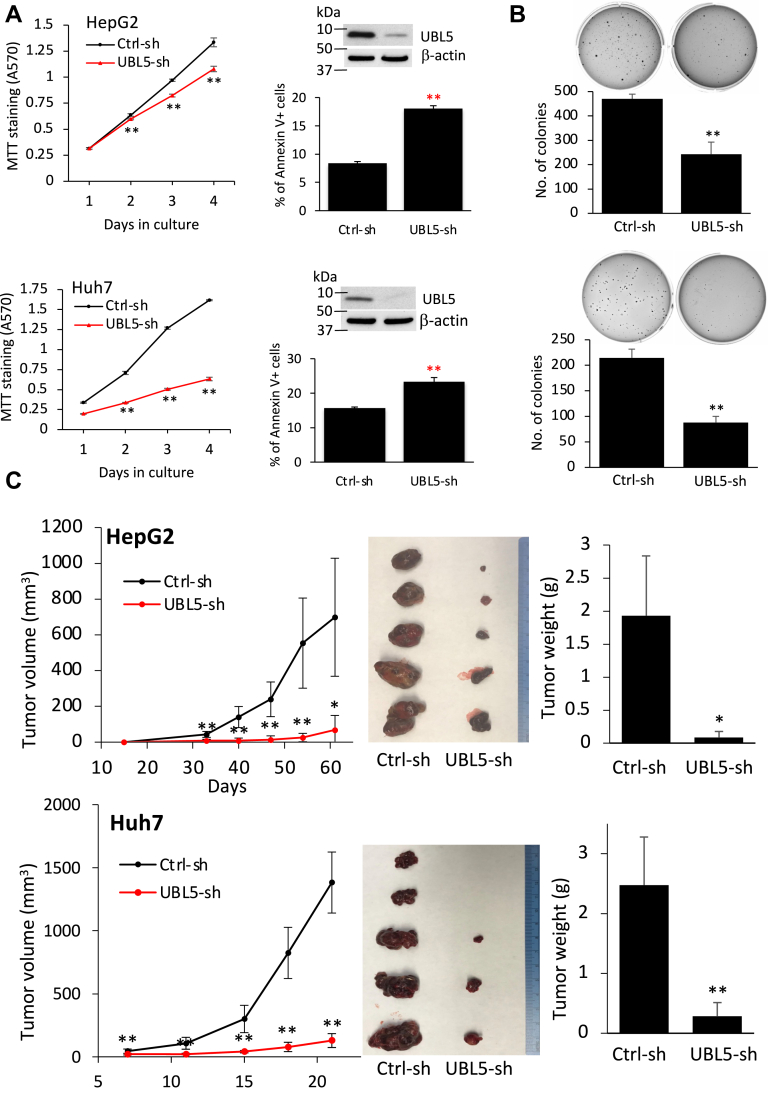
**UBL5 stable KD inhibits cell survival and tumorigenicity of cancer cells *in vivo*.***A*, expression of UBL5 in HepG2 and Huh7 cells was silenced by UBL5 shRNA. The pooled colonies of puromycin-resistant cells were established as continuous culture. The viability of the cells was analyzed by MTT. The apoptosis of the cells in culture for 2 days was then determined by Annexin V staining. The stable KD of UBL5 protein expression in these cells was confirmed by immunoblotting. *B*, the ability of the UBL5 shRNA and Ctrl-shRNA transduced cells to form colonies in anchorage-independent conditions was assessed by growing them in soft agar. The colonies formed in soft agar were stained, photographed, quantified, and presented as numbers of colonies/well. *C*, the UBL5 shRNA and Ctrl-shRNA-transduced HepG2 and Huh7 cells were s.c. injected in male NSG mice (n = 5). Tumor growth curves were constructed from tumor volumes measured at indicated times (days) postinoculation (*left*). Tumors of each group were excised and photographed at the endpoint when the animals were sacrificed (*middle*). Shown in *right* were comparisons of endpoint tumor weights between the UBL5 KD and the corresponding control group. KD, knockdown; MTT, 3-(4,5-dimethylthiazol-2-yl)-2,5-diphenyltetrazolium bromide; NSG, NOD scid gamma; UBL5, ubiquitin-like protein 5.

We next turned to a stable KD approach using virally transduced shRNA. The pooled colonies of puromycin-resistant cells were established as continuous culture while UBL5 remained to be silenced (Fig. 5*A*), likely as a result of recovery from or adaptation to the loss of UBL5. In early passages, these UBL5 shRNA KD cells showed reduced viabilities and more apoptosis (Fig. 5*A*). When plated in semisolid soft agar, these stable UBL5 KD cells were poor in forming colonies in this challenging anchorage-independent condition (Fig. 5*B*). Furthermore, the stable UBL5 KD cells became poorly tumorigenic after implantation to immunodeficient NOD scid gamma mice compared with the Ctrl-shRNA-transduced cells (Fig. 5*C*), indicating that proper expression of UBL5 is required for the survival of these tumor cells *in vivo*. Similar results were obtained from UBL5 KD SKOV-3 cells (Fig. S4, *A* and *B*). These results establish that the endogenous UBL5 is required to maintain cell survival *in vitro* and *in vivo*.

### Loss of UBL5 underlies ER stress–induced apoptosis

**Figure 6:**
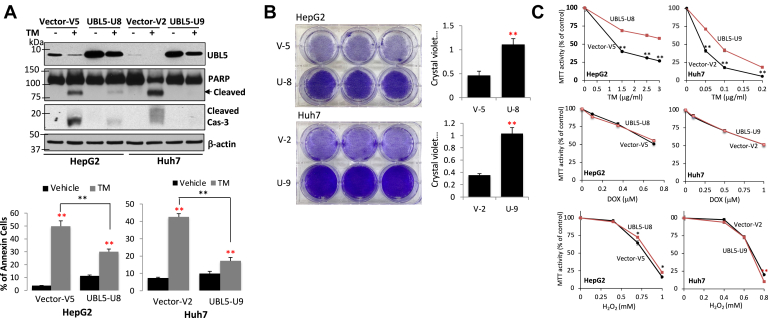
**Depletion of UBL5 underlies ER stress–induced apoptosis.***A*, HepG2 and Huh7 clones expressing exogenous UBL5 and vector control clones were treated for 3 days with TM at doses described for Figure 1*A*. Overexpression of UBL5, partial depletion of UBL5 by TM treatment, and prevention of PARP and Cas-3 cleavages in UBL5-overexpressing cells were confirmed by immunoblotting (*upper*). The percentages of apoptosis in these cells were analyzed by Annexin V staining (*lower*). *B*, UBL5-overexpressing and control cells were treated with TM for two (HepG2) or three cycles (Huh7). Remaining cells in culture plates were stained with crystal violet, and the staining intensities were quantified. *C*, UBL5-overexpressing and control cells were incubated with the indicated doses of TM as in (*A*), and cell viabilities were measured with MTT and presented as percent of activity relative to vehicle control (defined as 100%). As control experiments, these cells were also treated with other death stimuli DOX and H_2_O_2_ at the indicated concentrations to monitor cell viabilities with MTT assay. DOX, doxorubicin; ER, endoplasmic reticulum; H_2_O_2_, hydrogen peroxide; MTT, 3-(4,5-dimethylthiazol-2-yl)-2,5-diphenyltetrazolium bromide; PARP, poly(ADP-ribose) polymerase; TM, tunicamycin; UBL5, ubiquitin-like protein 5.

Unmitigated ER stress is known to induce apoptosis (41, 42). Given the prominent role of UBL5 in protection of cell survival and the regulatory mechanism to control the protein stability by the UPR–PERK arm, one would anticipate that PERK-mediated degradation of UBL5 will contribute to cell death from chronic or repeated ER stress. To address this possibility, we generated stable UBL5-overexpressing clones in HepG2 and Huh7 cells. Because of the high levels of UBL5 expression, the ER stressor TM induced only partial loss of the total UBL5 protein in these cells (Fig. 6*A*). The leftover UBL5 following TM treatment remained much higher than the endogenous UBL5 protein in untreated wt cells or vector-control cells. Thus, these UBL5-overexpressing cells provided an ideal tool to assess the death response to ER stress in the presence of unregulated UBL5 component. Indeed, TM stimulated significant apoptosis as indicated by induction of cleavage of poly(ADP-ribose) polymerase or Cas-3 and Annexin V+ populations, which was partially prevented by overexpression of UBL5 (Fig. 6*A*).

To further explore the rescuing effect of UBL5, the UBL5-overexpressing and control cells were exposed to repeated cycles of TM at a modest dose with a 24 h recovery between TM treatment. After two and three cycles in HepG2 and Huh7 cells, respectively, the control cells experienced dramatic loss in density, whereas UBL5-overxpressing cells survived the repeated treatment with TM and reached confluence in culture (Fig. 6*B*). We also examined the TM dose responses in these cells. As shown in Figure 6*C*, treatment with TM for 3 days reduced survivability in a dose-dependent manner. As reflected by the right shift of the response curves, overexpression of UBL5 rendered these cells more resistant to ER stress–induced cell death (Fig. 6*C*). Similar results were observed from independent UBL5-overexpressing clones. In contrast to TM treatment, UBL5 overexpression did not confer resistance to DOX or H_2_O_2_ (Fig. 6*C*), death insults that did not disrupt UBL5 protein stability (Fig. 1*B*).

## Discussion

UBL5 has been viewed as a mitochondrial stress gene mainly based on investigations in *C. elegans*, a model organism for the study of the UPR^mt^ (15, 43). A systematic RNAi inactivation approach identified a role for UBL5 in induction of the mitochondrial chaperones hsp-60 during the UPR^mt^ (16). A UBL5 transgene under the endogenous *ubl5* promoter was induced and enriched in the nucleus where it complexed with the transcription factor Dve-1 to activate expression of hsp-60 (17). Given the centrality of the ER in overall protein folding, maturing, and trafficking, the ER stress-UPR is physiologically more prevailing and functionally more important in the mammalian system (44). Furthermore, the ER-UPR differs from the UPR^mt^ in causes, signaling networks, target genes, and functional outcomes (44, 45, 46), making it unlikely that UBL5 plays a common role under these stress settings.

**Figure 7:**
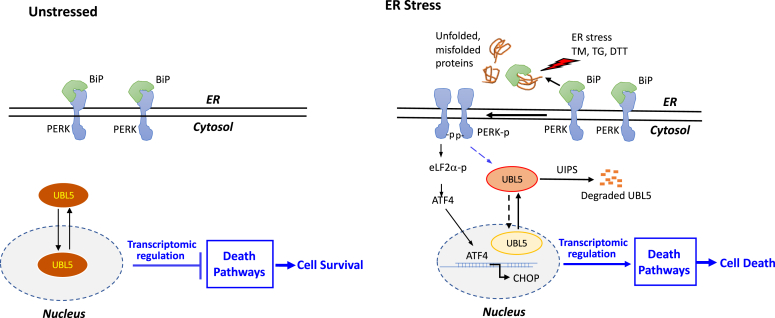
**Summary of the signaling network from ER stress to inhibition of UBL5 protein stability and prosurvival function.** In unstressed state, UBL5 protein is stable and involved in suppression of multiple death pathways contributing to cell survival. During ER stress, the UPR–PERK axis induces proteolytic degradation of UBL5 protein *via* UIPS, leading to the loss of UBL5 death-suppressive function, a proapoptotic mechanism in parallel with the ER stress–ATF4–CHOP death pathway. ATF4, activating transcription factor 4; CHOP, C/EBP homologous protein; ER, endoplasmic reticulum; UBL5, ubiquitin-like protein 5; UIPS, ubiquitin-independent proteasome system.

We therefore examined whether UBL5 is physiologically regulated and participates in the ER stress response. Our studies demonstrated that UBL5 underwent rapid depletion in response to various pharmacological ER stressors and other indirect stimuli. The loss of UBL5 protein was mediated by UIPS. UBL5 degradation occurred downstream of PERK activation. In agreement with this location of UBL5 in the UPR signaling, the lack of UBL5 did not affect activation of the three UPR arms in response to ER stress (Fig. S5). These results represent the only known mechanism to regulate UBL5 protein stability, the first time to link the ER stress-UPR signaling to the control of UBL5 activity (Fig. 7).

Although UBL5 KD has been shown to induce apoptosis in cancer cell lines (10, 12), little is known about the underlying mechanisms and physiological relevance. There is no biochemical basis for UBL5 protein to act as an intrinsic antiapoptotic mediator such as Bcl-2. Most likely, UBL5 exerts its effect through regulating transcriptional programs to balance death and survival signals. The loss of UBL5 function would disrupt the balance to favor cell death. Indeed, a plethora of well-defined death pathways were systematically activated in UBL5 KD cells as revealed by RNA-Seq and pathway enrichment analysis. Furthermore, the phenotypes of UBL5-deficient cells in culture and immunodeficient mice confirmed that UBL5 is required to maintain cell viability *in vitro* and *in vivo*.

The evolutionally conserved UPR is activated upon protein overloading to restore ER homeostasis and survival of cells (46). However, excessive or prolonged activation of the UPR is known to trigger apoptosis to eliminate overstressed cells (41, 42, 47). Each of the three UPR arms is theoretically linked to activation of the intrinsic and extrinsic pathways of apoptosis. These proapoptotic effectors include CHOP, the tumor necrosis factor receptor–associated factor 2 activated by IRE1, and Bax/Bcl2-regulated Ca^2+^ release from the ER (41, 42, 47). Among them, the proapoptotic transcriptional factor CHOP seems to play a central role. Its target genes include GADD34, DR5 (TRAIL receptor-2), and Ero1α (ER oxidoreductase-1) (41, 42, 47). However, CHOP is commonly induced under ER stress in a sustainable manner, which is not always associated with apoptotic induction, implying additional mechanisms in turning the UPR to a marked death response. The enhanced resistance of UBL5-overexpressing cells to TM indicates that PERK-mediated loss of UBL5 protein is another significant contributor to the death response to ER stress independent of the ATF4–CHOP cascade as illustrated in Figure 7. When we treated HepG2 and Huh7 cells with TM, severe apoptosis was observed after 3 days of exposure, a time course in agreement with a causal role of UBL5 depletion in activation of death signaling cascades and apoptosis. Notably, UBL5 overexpression did not protect against other death stimuli that did not disrupt UBL5 protein, suggesting that the endogenous level of UBL5 is critical and sufficient for survival protection. Overexpression of UBL5 provides a survival advantage only when the endogenous UBL5 protein is impaired.

In the present work, we showed the presence of cellular UBL5 protein in both cytosol and the nuclei of mammalian cells, consistent with the control of its stability in the cytosol by the UIPS and its potential function in the nucleus to impact gene expression. UBL5 may serve as a cofactor to certain transcription factors such as SATB2, the mammalian ortholog of Dve-1 (17). Interestingly, SATB2 is a global chromatin organizer that could have a genome-wide impact on gene expression (48). A physical binding of UBL5 with SATB2 was observed only when they were ectopically overexpressed in 293T cells (17). In an effort to identify the molecular mechanism for UBL5 regulation of gene expression, we were unfortunately unable to detect UBL5 binding with SATB2 through coimmunoprecipitation analysis (data not shown).

As another possibility, UBL5 may affect maturation of protein-coding transcripts *via* regulating pre-mRNA splicing. Of note, UBL5 has been shown to bind with SART1 (the homolog of the yeast Snu66) (9, 10, 11, 12) and is copresent with components of the pre-mRNA spliceosome (11, 39). A genome-wide screen with RNA-Seq in HeLa cells showed that UBL5 depletion led to decreased pre-mRNA splicing efficiency and globally enhanced intron retention (11). Based on this scenario, general downregulation of most transcripts and corresponding proteins is expected for UBL5-deficient cells. However, we observed both upregulated and downregulated gene sets responsive to UBL5 KD (Fig. S6*A*). Usually transcripts of large genes are more prone to the defective mRNA splicing. For instance, the mega *FASN* (fatty acid synthase) has been commonly used as an example to examine defective mRNA splicing and intron retention (11). When we examined abundances of various *FASN* transcripts from the RNA-Seq data, we indeed observed that two intronic transcripts were significantly increased in UBL5 siRNA KD compared with control cells (Fig. S6*B*). However, the major *FASN* protein–coding transcript was also upregulated by UBL5 KD (Fig. S6*B*). Overall, the FASN protein was moderately increased in UBL5 KD cells (Fig. S6*C*). Apparently, the complex effects of UBL5 depletion on gene expression involve more complicated mechanisms than general interference with pre-mRNA splicing. Our results are also consistent with the fact that only a few genes, including Mcl-1 (12), Sororin, LZTS2, and XRCC3 (11), have been shown so far to be downregulated on protein levels by UBL5 depletion through this proposed pre-mRNA splicing mechanism.

A remarkable observation from the present work is the specific role of PERK in the regulation of UBL5 turnover. Our results indicate that UBL5 stability is controlled exclusively by PERK activity independent of other redundant or adaptive regulators. The current literature on PERK and UBL5 does not offer any biochemical ground for their physical interactions or PERK direct phosphorylation of UBL5. UBL5 protein does not appear to be phosphorylated in serine or threonine residues (as judged by mobility) in resting or ER-stressed cells. Furthermore, UBL5 and PERK did not coimmunoprecipitate each other in these conditions (data not shown).

UBL5 is not the only protein to be degraded in a PERK-dependent manner. Cyclin D1 and the tumor suppressor protein p53 get degraded upon activation of PERK under ER stress to contribute to cell cycle arrest and the inhibition of apoptosis, respectively (49, 50, 51). PERK does not directly phosphorylate p53 or cyclin D1. The regulation of p53 seems to involve PERK phosphorylation of glycogen synthase kinase 3β as an intermediate kinase that, in turn, induces phosphorylation and proteasome degradation of p53 (49, 50). It is possible that a common mechanism might be involved in PERK-dependent proteasomal degradation of these proteins under ER stress.

Our study identifies UBL5 as a new member of UIPS-regulated proteins. While most cellular proteins are degraded *via* ubiquitin proteasome system, an increasing number of proteins are now known to be removed by the route of UIPS. The prototype examples are ornithine decarboxylase, thymidylate synthase, and Rpn4 (19, 52). However, very diverse mechanisms are involved in degradation of these UIPS targets. Little can be inferred from these well-studied examples to predict the specific proteasome, activator, and the essential amino acid sequence (degron) involved in the degradation of UBL5. There are two possibilities to activate UBL5 degradation by UIPS in response to ER stress. First, PERK activation could induce change(s) in UBL5 structure, localization, or interaction with other proteins to render it more sensitive or more accessible to the UIPS activity. Alternatively, PERK activation could increase cellular UIPS activity toward its target proteins. Of note, overexpressed UBL5 protein was only partially degraded in response to ER stress. The observation suggests that the UIPS activity in ER-stressed cells is tuned to degrade physiological levels of UBL5 protein. It will be interesting to determine how PERK communicates with UBL5 to tightly control UBL5 stability/function and cell fate.

## Experimental procedures

### Reagents

Cell culture media, PBS, antibiotics, DTT, Lipofectamine 2000, Lipofectamine 3000, Lipofectamine RNAimax, MTT, Trizol, the Pierce BCA protein assay kit, the enhanced chemiluminescence detection kit, the Phusion site-directed mutagenesis kit, the high-capacity complementary DNA (cDNA) RT kit, the TaqMan universal PCR master mix, human UBL5 siRNA (4392422 s224520), nontarget control siRNA (AM4635), and quantitative PCR probes for UBL5, CHOP, and β-actin were obtained from ThermoFisher Scientific. Fetal bovine serum was from Atlanta Biologicals. The protease/phosphatase inhibitor cocktail was from Roche Diagnostics. The RNeasy Mini Kit and Endo-Free Plasmid Maxi Kit were purchased from Qiagen. Tricine, TM, TG, rotenone, metformin, piericidin A, DOX, cisplatin, bafilomycin A1, ceapin-A7, CCT020312, and BODIPY 493/503 were purchased from Sigma–Aldrich. MG-132 and 3-methyladenine were purchased from Selleck Chemicals. GSK2656157 and STF083010 were purchased from ApexBio Technology. H_2_O_2_ was from Fisher Chemical.

The antibodies against the following proteins were from Cell Signaling: binding immunoglobulin protein (catalog no.: 3177), phosphorylated eIF2α (catalog no.: 3398), eIF2α (catalog no.: 5324), CHOP (catalog no.: 2895), phospho-c-Jun N-terminal kinase (catalog no.: 9255), phospho-AMP-activated kinase α (catalog no.: 2535), phospho-PERK (catalog no.: 3179), PERK (catalog no.: 5683), IRE1α (catalog no.: 3294), ATF4 (catalog no.: 11815), LC3 I/II (catalog no.: 12741), Lamin A/C (catalog no.: 2032), poly(ADP-ribose) polymerase (catalog no.: 9542), cleaved-caspase3 (catalog no.: 9661), FASN (catalog no.: 3180), GAPDH (catalog no.: 5174), β-actin (catalog no.: 4970), and the horseradish peroxidase–conjugated secondary antibodies (catalog nos.: 7074 and 7076). The anti-UBL5 antibody (catalog no.: PA5-70203) was obtained from ThermoFisher Scientific. The anti-phosphorylated IRE1α antibody was from Novus Biologicals (catalog no.: NB100-2323). The antibodies against ATF6 (catalog no.: ab122897), goat anti-rabbit IgG H&L (Alexa Fluor 488) (catalog no.: ab150077), and goat antimouse IgG H&L (Alexa Fluor 594) (catalog no.: ab150116) were purchased from Abcam. The anti-c-Myc antibody was obtained from Santa Cruz Biotechnology.

### Cells

The hepatocellular carcinoma cell lines HepG2 and Huh7, the ovarian cancer cell line SKOV-3, and the embryonic kidney cell line 293TN were originally obtained from American Type Culture Collection. These cell lines were cultured in low-glucose Dulbecco's modified Eagle's medium (HepG2 and Hul7), RPMI1640 (SKOV-3), and high-glucose Dulbecco's modified Eagle's medium (293TN) supplemented with 10% fetal bovine serum and antibiotics. The cells were frozen at early passages and used for less than 1 month in continuous culture.

### Modulation of gene expression

The human UBL5 cDNA (GenBank accession no.: NM 001048241.3) was amplified by PCR and cloned into the pCDH-CMV retroviral vector (System Biosciences) to make pCDH-CMV-UBL5. The nine lysine residues (K12, K13, K17, K28, K29, K41, K45, and K46) were mutated to arginine (R) individually or collectively to generate the indicated mutants. The human PERK expression vector pCDNA3-PERK was obtained from Addgene #21814. The pCDNA3-PERK K618A was prepared by site-directed mutagenesis from pCDNA3-PERK (37). Plasmid DNAs were purified using the endo-free purification kit, and DNA sequences of all plasmids were verified by automatic sequencing.

The shRNA target sequences (control shRNA: 5′-CCTAAGGTTAAGTCGCCCTCG-3′, UBL5 shRNA58: 5′-CCTGGAGCTTTATTATC-3′, PERK shRNA: 5′-GGAACGACCTGAAGCTATAAA-3′) were cloned into the shRNA lentiviral vector pGreenPuro (System Biosciences). The guide RNA sequences to KO PERK (5′-GAATGGACGATGTACCATAGAGG-3′) were cloned into the lentiCRISPRv2 vector (Addgene plasmid #52961) (53). The aforementioned reagent and resource table listed all siRNA, shRNA, and guide RNA sequences used in the study. The recombinant lentivirus was produced in 293TN cells by cotransfection with lentiviral vectors and packaging plasmids using Lipofectamine 2000. The culture supernatants were collected 48 to 72 h post-transfection and used for infection of cells in culture. The transduced cells were selected with puromycin to establish stably transduced populations or clones.

### RT and real-time PCR

Total cellular RNA was extracted with Trizol from cells in culture. Residual genomic DNA was removed by treatment with DNA-free DNase kit. cDNA was synthesized using the High-Capacity cDNA RT Kit. The cDNA abundances of UBL5 and β-actin were quantified using gene-specific probes, the TaqMan Universal PCR Master Mix, and the Bio-Rad CFX Connect Real-Time PCR System. The results of UBL5 mRNA expression were normalized to the level of β-actin and presented as fold changes relative to that of untreated control cells (defined as 1).

### Immunoblotting analysis

Cells were lysed with SDS sample buffer or a lysis buffer containing 40 mM Hepes (pH 7.4), 150 mM NaCl, 1% Triton, 0.5% sodium deoxycholate, 0.1% SDS, 10 mM NaF, 5 mM sodium pyrophosphate, 1 mM β-glycerophosphate, 1 mM NaV, and the protease and phosphatase inhibitor cocktail. Protein concentrations were quantified with the Pierce BCA Protein Assay Kit. Equal amounts of proteins were resolved by SDS-PAGE. Tricine–SDS-PAGE (16% acrylamide in running gel) (54) instead of regular SDS-PAGE was used to analyze the small UBL5 protein. Proteins on gels were transferred to immunoblot polyvinylidene difluoride membranes and immunoblotted following the protocols of the manufacturers of primary antibodies. Immunocomplexes were visualized by an enhanced chemiluminescence detection kit using the horseradish peroxidase–conjugated secondary antibodies.

### Immunofluorescence staining

Cells were seeded in the Nunc glass bottom dishes and fixed with −20 °C prechilled methanol for 10 min. After blocking for 1 h with 1.5% goat serum containing 1% of bovine serum albumin, cells were incubated with anti-UBL5 antibody (1:100 dilution) overnight followed by the Alexa488- and Alexa594-conjugated secondary antibodies (1:500 dilution) for 1 h. The glass slips were mounted with the ProLong Gold Antifade Mountant containing 4′,6-diamidino-2-phenylindole. The staining and subcellular localizations were examined with all-in-one fluorescence microscope BZ-X800.

### Cellular fractionation

Cellular nuclei were isolated from the cytosol essentially according to Schreiber *et al.* (55). Briefly, HepG2 and Huh7 cells were resuspended in a cold hypotonic lysis buffer (20 mM Hepes [pH 7.5], 10 mM KCl, 3 mM MgCl_2_, 1 mM NaV, and 1× protease/phosphatase inhibitor mix) and incubated on ice for 15 min. NP-40 was added to a final concentration of 0.5%. After vortex for 10 s, the homogenates were incubated on ice for 1 min and centrifuged at 3000 rpm at 4 °C for 4 min. The supernatants were collected as the cytoplasmic fraction. The nuclear pellet was washed one time with the hypotonic lysis buffer containing 0.5% NP-40 and then solubilized immediately in 1× SDS sample buffer.

### RNA-Seq

Approximately 0.5 μg total RNA was used for RNA-Seq library preparation by following the Illumina TruSeq stranded mRNA sample preparation guide. The first step in the workflow involved purifying the poly-A-containing mRNA molecules using poly-T oligo-attached magnetic beads. The mRNA was fragmented into small pieces using divalent cations under elevated temperature. The cleaved RNA fragments were copied into first-strand cDNA using reverse transcriptase and random primers, followed by second-strand cDNA synthesis using DNA polymerase I and RNase H. Strand specificity was achieved by replacing dTTP with dUTP in the second-strand marking mix. These cDNA fragments went through an end repair process, the addition of a single “A” base, and then ligation of the adapters. The products were purified and enriched with PCR to create the final RNA-Seq library. RNA-Seq libraries were subjected to quantification process, pooled for cBot amplification, and subsequent 50 bp single-read sequencing run with Illumina HiSeq 3000 platform.

After the sequencing run, demultiplexing with Bcl2fastq2 was employed to generate the fastq file for each sample. The average of 30 M reads was obtained for this set of samples. The quality of RNA-Seq reads was assessed with FastQC, version 0.11.9 (https://www.bioinformatics.babraham.ac.uk/projects/fastqc/, https://www.bibsonomy.org/bibtex/f230a919c34360709aa298734d63dca3, Accessed June 26, 2022). The reads were aligned using STAR aligner (56) version 2.7.6a to reference genome GRCh38. Raw gene counts of mapped reads were aggregated using featureCounts (57). The differential gene expression analysis was performed with Bioconductor package DESeq2, version 1.30.0 (58) using the normalized and filtered counts per gene from the RNA-Seq data. Differentially expressed genes with adjusted *p* value <0.05 were subject to functional annotation and pathway enrichment analyses using the DAVID (59) and ingenuity pathway analysis programs.

### Cell viability and apoptosis assays

Cells were seeded in complete medium in 6- or 12-well plates. Following treatments with indicated compounds or vehicle, cells were stained with MTT assay (40) or crystal violet staining (60). The MTT or crystal violet intensity curves were plotted as a function of exposure days or drug doses used in the experiments. Cell numbers were quantified with the Beckman Coulter Counter.

The ability of cells to grow in anchorage-independent conditions was assessed by growing cells in complete medium containing 0.3% soft agar. Six-well plates were first coated with 1.5 ml complete medium with 0.6% soft agar to prevent attachment of cells to the bottoms of the wells. Cells (5000/well) in complete medium containing 0.3% soft agar were overlayered onto the precoated wells and incubated for 3 and 2 weeks for HepG2 and Huh7, respectively. Fresh 100 μl of growth medium was applied to the top twice weekly. At the end of the experiment, colonies in soft agar were stained with crystal violet and photographed by using the Bio-Rad ChemiDoc Imaging Systems. The colonies larger than 100 μm in diameter were quantified from triplicate wells.

The FITC Annexin V Apoptosis Detection Kit was obtained from BioLegend. Following the indicated treatments, cells were stained with FITC Annexin V and propidium iodide. The fluorescence signals were measured using BD FACS Canto flow cytometer (BD Biosciences). The results were analyzed with FACs DIVA software (BD Biosciences).

### Animal studies

All animal experiments were approved by the Virginia Commonwealth University Institutional Animal Care and Use Committee.

To determine the response of UBL5 to ER stress *in vivo*, 3-month-old C57BL/6 male mice (Charles River Labs) were i.p. injected with TM (2 mg/kg body weight) (61) or vehicle (0.5% methylcellulose). The mice were sacrificed 26 h postinjection. Fractions of liver tissues were homogenized for immunoblotting analysis.

To determine the effect of UBL5 KD *in vivo*, UBL5 shRNA-transduced and their nontarget control (ctrl-shRNA) cells were implanted to 8-week-old male NOD scid gamma mice. The cells in exponential growth phase were injected s.c. (4 × 10^6^ cells/0.1 ml) on the right flank. The formation of xenografts was monitored and measured with a digital caliper. The tumor volumes were calculated based on the formula lw^2^/2, where l was the length and w was the shortest width of the tumor.

### Statistics

All numerical data from *in vitro* studies were presented as mean ± SD of triplicate assays, representative of three independent experiments. The statistical significances were analyzed using Student's *t* test unless otherwise stated, where *p* <0.05 was considered statistically significant. In figures, the statistical significances were indicated with n.s. (not significant) if *p* >0.05, ∗ if *p* <0.05, or ∗∗ if *p* <0.01.

## Material and data availability

Experimental reagents generated in this study will be available upon request. The RNA-Seq data were submitted to National Center for Biotechnology Information Gene Expression Omnibus database: GSE209935 (https://www.ncbi.nlm.nih.gov/geo/query/acc.cgi?acc=GSE209935).

## Supporting information

This article contains supporting information.

## Conflict of interest

The authors declare that they have no conflicts of interest with the contents of this article.

## Supporting information

Supporting Figure S3

Supporting Figure S4A

Supporting Figure S4B
