# Supplementary material for: Ubiquitin-like protein 5 is a novel player in the UPR–PERK arm and ER stress–induced cell death
Source: J Biol Chem. 2023 Jun 12;299(7):104915. doi: 10.1016/j.jbc.2023.104915 (PMC10339194; doi:10.1016/j.jbc.2023.104915)
Supplement: Supporting Figure S1 [file mmc1.pdf]

Supplementary Figure S1

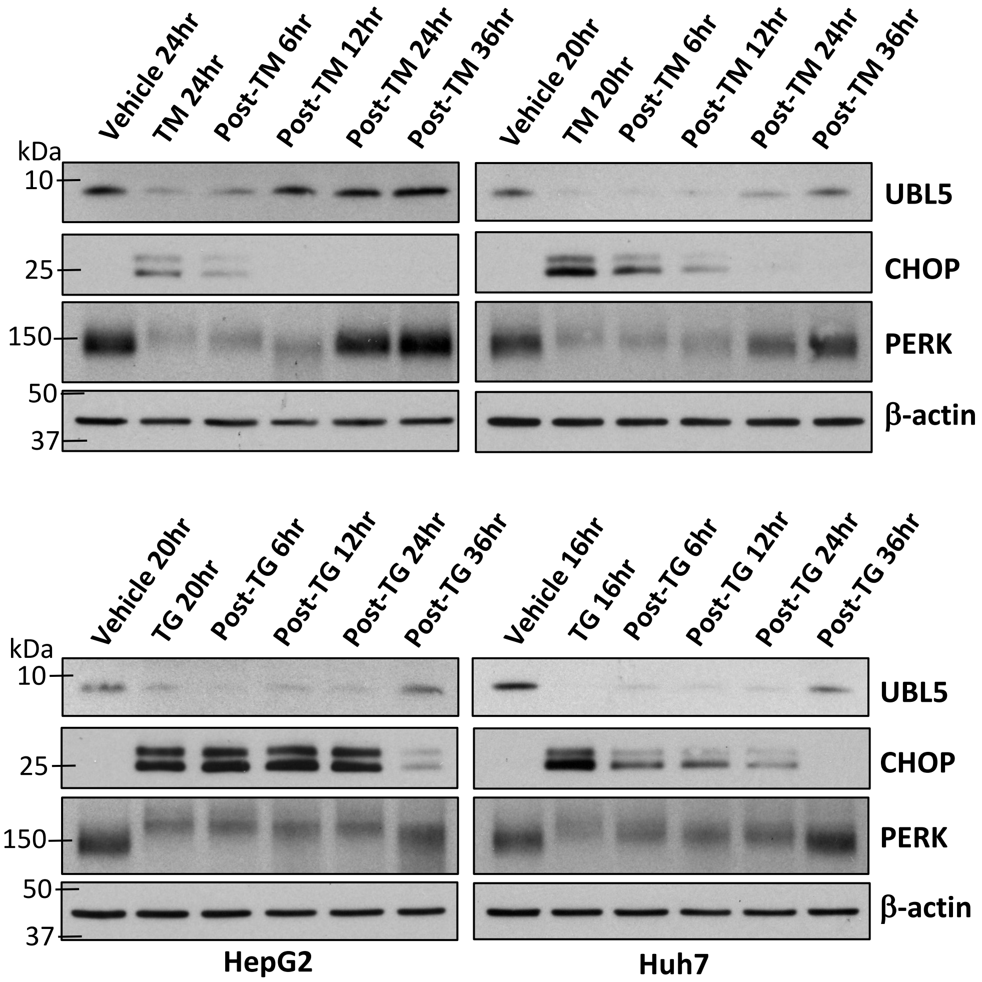

Figure S1. UBL5 recovers after complete clearance of ER stress. HepG2 and Huh7 cells were treated with TM or TG at doses as in Figure 1A for the indicated times (hr). After removal of TM or TG-containing medium, the cells were washed with PBS, and incubated with complete medium without TM or TG for the indicated times of recovery from ER stress. Expression of UBL5, CHOP induction or PERK phospho-associated mobility shift were examined by immunoblotting.
