# Supplementary material for: Ubiquitin-like protein 5 is a novel player in the UPR–PERK arm and ER stress–induced cell death
Source: J Biol Chem. 2023 Jun 12;299(7):104915. doi: 10.1016/j.jbc.2023.104915 (PMC10339194; doi:10.1016/j.jbc.2023.104915)
Supplement: Supporting Figure S2 [file mmc2.pdf]

Supplementary Figure S2

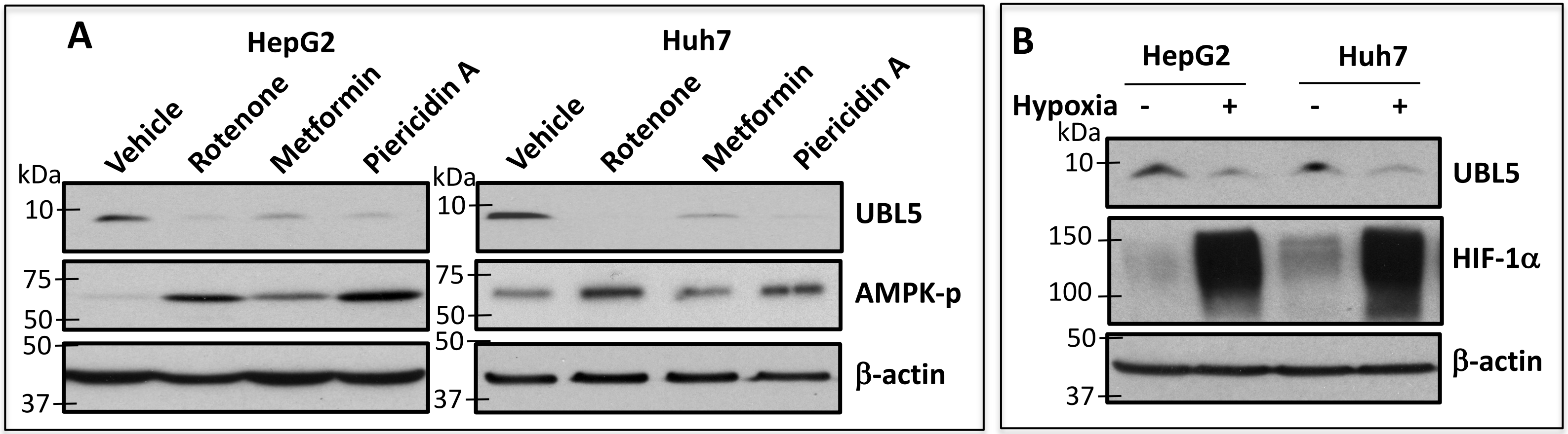

Figure S2. Indirect stimuli of ER stress also induce UBL5 depletion. ***A.*** HepG2 and Huh7 cells were treated for 24 hours with the ETC inhibitor Rotenone (0.33 μM), Metformin (2 mM), or Piericidin A (25 nM). Expression of UBL5 and AMPK-p (marker of energy deficiency) was examined by immunoblotting. ***B***. HepG2 and Huh7 cells were incubated at 37 °C for 18 hours in a hypoxia chamber with 1% O_2 and 5% CO_2 balanced with N_2 or in a control chamber with air and 5% CO_2. Expression of UBL5 and HIF-1α (hypoxia marker) was examined by immunoblotting.
