# Supplementary material for: Ubiquitin-like protein 5 is a novel player in the UPR–PERK arm and ER stress–induced cell death
Source: J Biol Chem. 2023 Jun 12;299(7):104915. doi: 10.1016/j.jbc.2023.104915 (PMC10339194; doi:10.1016/j.jbc.2023.104915)
Supplement: Supporting Figure S3 [file mmc3.pdf]

Supplementary Figure S3

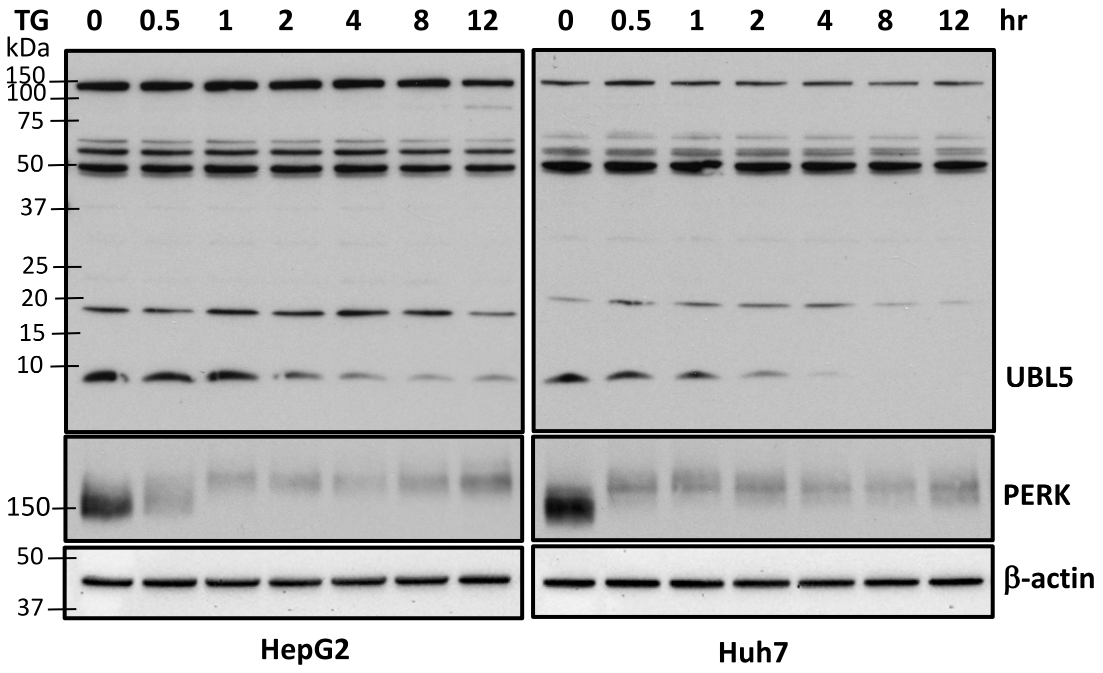

Figure S3. ER stress-induced UBL5 depletion is not associated with formation of UBL5-ubiquitin ladders. HepG2 and Huh7 cells were treated with TG at doses as in Figure 1A for the indicated times (hr). Expression of UBL5, PERK and β-actin was examined by immunoblotting.
