# Supplementary figures and images for: Ubiquitin-like protein 5 is a novel player in the UPR–PERK arm and ER stress–induced cell death
Source: J Biol Chem. 2023 Jun 12;299(7):104915. doi: 10.1016/j.jbc.2023.104915 (PMC10339194; doi:10.1016/j.jbc.2023.104915)

## Supplementary Figure S4A

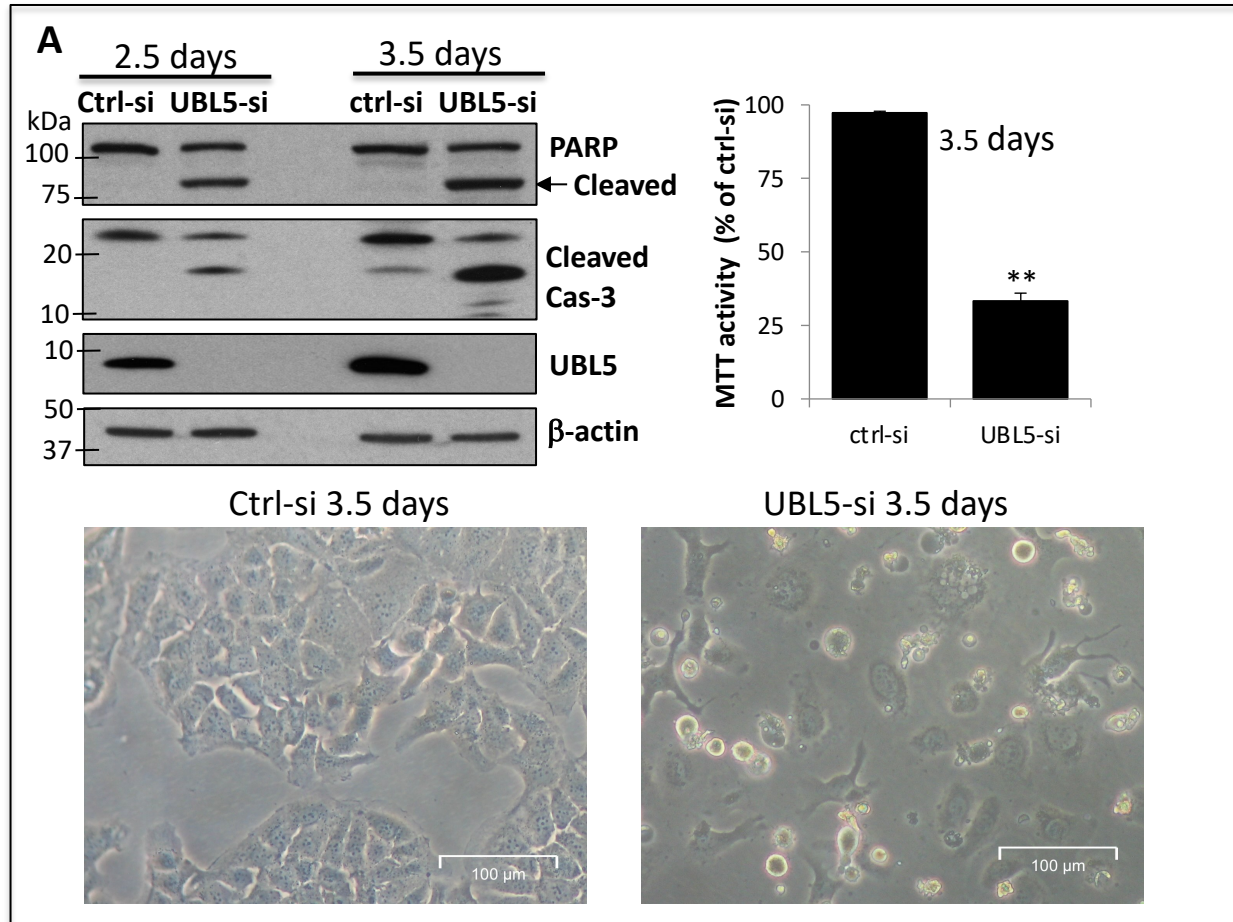

Supplement: Supporting Figure S4A [file mmc4.pdf]
