# Supplementary material for: Ubiquitin-like protein 5 is a novel player in the UPR–PERK arm and ER stress–induced cell death
Source: J Biol Chem. 2023 Jun 12;299(7):104915. doi: 10.1016/j.jbc.2023.104915 (PMC10339194; doi:10.1016/j.jbc.2023.104915)
Supplement: Supporting Figure S4B [file mmc5.pdf]

Supplementary Figure S4B

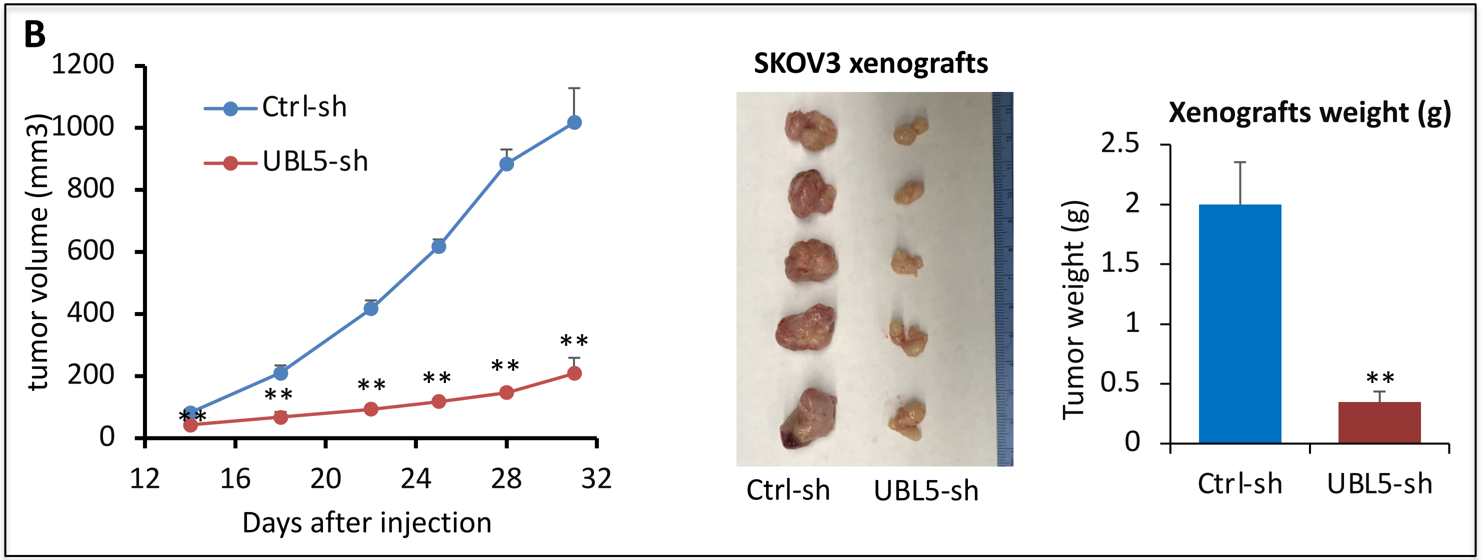

Figure S4. **UBL5 KD inhibits survival of SKOV-3 cells in culture and tumorigenicity in mice.** ***A.*** SKOV-3 cells were transfected with Ctrl-si or UBL5 siRNA. Shown were expression of apoptotic markers (***upper left***), cell viability by MTT staining (***upper right***), and phase-contrast photographs of ctrl-si and UBL5 siRNA transfected cells (***lower***). ***B.*** UBL5 shRNA inhibited tumorigenicity of SKOV-3 cells in mice. The Ctrl-sh and UBL5 shRNA KD SKOV-3 cells were established as continuous culture. After confirmation of stable KD efficiency, the cells ($4x10^6$/mouse) were s.c. injected in 8-week old female NSG mice. Tumor growth curves were constructed from tumor volumes measured at indicated times (days) post cell inoculation (***left***). Xenografts from each group were excised and photographed at the endpoint (***middle***). Shown in ***right*** was comparison of endpoint tumor weights between Ctrl-sh and UBL5-shRNA knockdown groups.
