# Supplementary material for: Ubiquitin-like protein 5 is a novel player in the UPR–PERK arm and ER stress–induced cell death
Source: J Biol Chem. 2023 Jun 12;299(7):104915. doi: 10.1016/j.jbc.2023.104915 (PMC10339194; doi:10.1016/j.jbc.2023.104915)
Supplement: Supporting Figure S5 [file mmc6.pdf]

Supplementary Figure S5

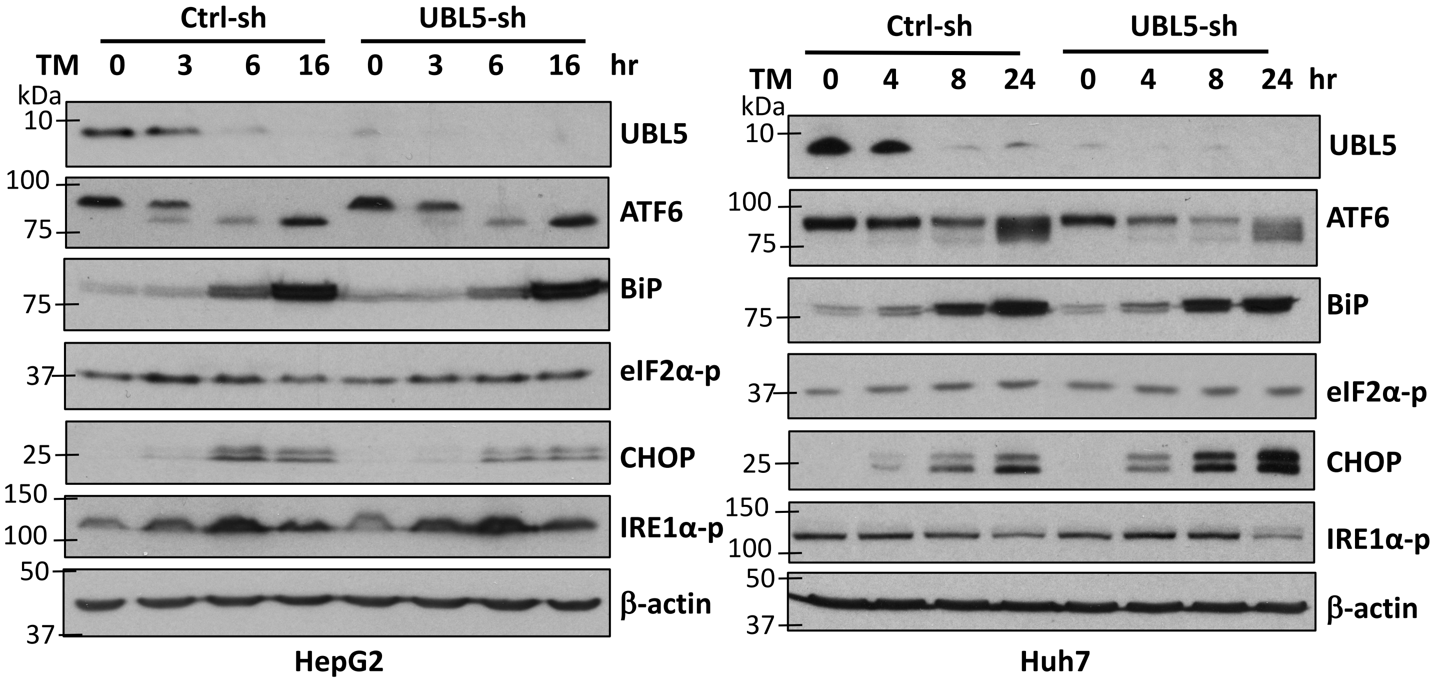

Figure S5. UBL5 KD does not affect activation of the UPR signaling. HepG2 and Huh7 cells were treated with TM at doses as in Figure 1A for the indicated time (hr). Expression of UBL5 and UPR activation were examined by immunoblotting analysis showing that multiple markers of the 3 arms of the UPR were equally activated in both Ctrl-shRNA and UBL5 shRNA KD cells.
