# Supplementary material for: Ubiquitin-like protein 5 is a novel player in the UPR–PERK arm and ER stress–induced cell death
Source: J Biol Chem. 2023 Jun 12;299(7):104915. doi: 10.1016/j.jbc.2023.104915 (PMC10339194; doi:10.1016/j.jbc.2023.104915)
Supplement: Supporting Figure S6 [file mmc7.pdf]

Supplementary Figure S6

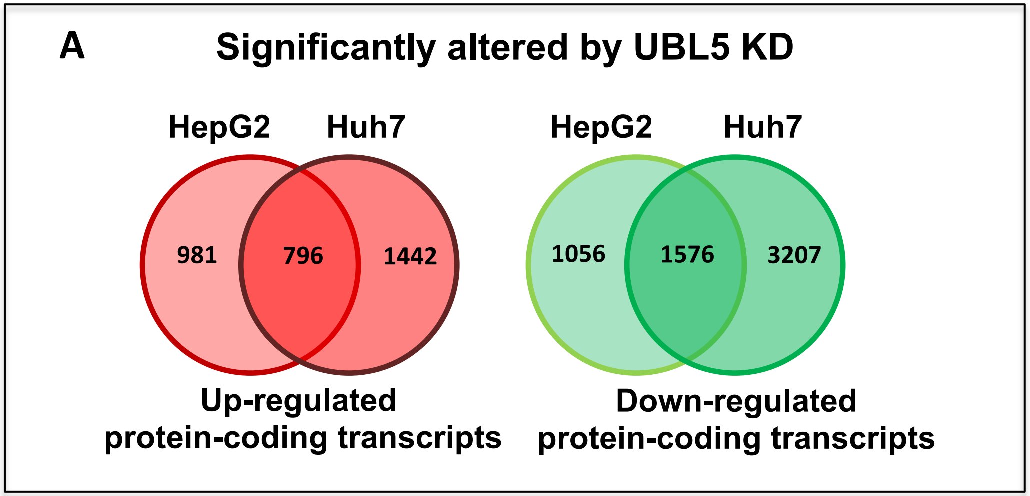

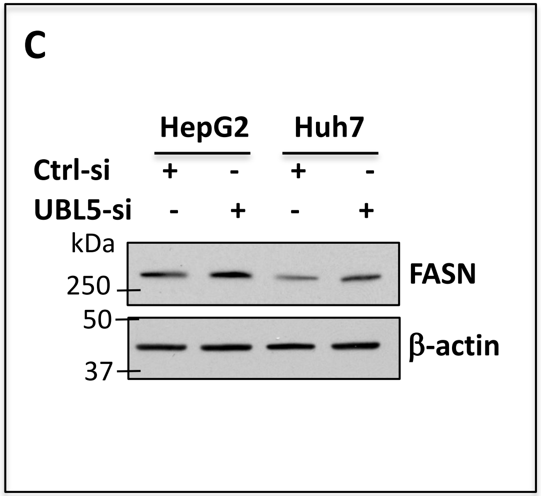

B

Expression of FASN transcripts

gene_name	ensembl_transcript_id	transcript_type	HepG2 Ctrl-si	HepG2 UBL5-si	HepG2 padj	Huh7 Ctrl-si	Huh7 UBL5-si	Huh7 padj
FASN	ENST00000306749	protein coding	0	0.6268	3.17E-02*	0	0.4338	4.09E-04**
FASN	ENST00000637026	protein coding	0	0.2936	9.54E-01n.s.	0	0.0941	9.78E-01n.s.
FASN	ENST00000635197	protein coding	0	-0.2627	7.72E-01n.s.	0	0.0071	9.94E-01n.s.
FASN	ENST00000584610	intron retention	0	2.4688	1.87E-08**	0	3.1192	3.59E-44**
FASN	ENST00000579410	intron retention	0	6.4123	7.39E-04**	0	3.8069	1.01E-08**
FASN	ENST00000578424	intron retention	0	4.5234	1.47E-02*	0	1.8709	8.67E-02n.s.
FASN	ENST00000580382	nonsense mediated decay	0	-0.9454	8.01E-01n.s.	0	-5.9279	1.63E-03**
FASN	ENST00000637525	processed transcript	0	3.9390	1.10E-01n.s.	0	1.9264	8.91E-02n.s.

log2FoldChange: -7, 0, 7

Figure S6. Effects of UBL5 siRNA KD on gene expression are more complicated than interference with mRNA splicing. ***A***. UBL5 siRNA KD regulated gene expression both positively and negatively. Shown were up- and down-regulated groups of genes and the overlaps between two cell lines. ***B***. UBL5 siRNA KD led to increases in 2 intronic transcripts and a protein-coding transcript of FASN in HepG2 and Hul7 cells. Expression of *FASN* transcripts were quantified from RNA-Seq data and presented as log2 fold changes. ***C***. Immunoblotting analysis was performed to show the effect of UBL5 siRNA KD on FASN protein expression.
